# Scintigraphic Imaging of Inflammation in the Aortic Wall Using ^99m^Tc-Pyrophosphate

**DOI:** 10.17691/stm2021.13.6.07

**Published:** 2021-12-28

**Authors:** J.N. Ilyushenkova, D.S. Panfilov, V.V. Saushkin, E.L. Sonduev, B.N. Kozlov, S.I. Sazonova

**Affiliations:** Senior Researcher, Nuclear Medicine Department; Cardiology Research Institute, Tomsk National Research Medical Center of the Russian Academy of Sciences, 111a Kievskaya St., Tomsk, 634012, Russia; Senior Researcher, Department of Cardiovascular Surgery; Cardiology Research Institute, Tomsk National Research Medical Center of the Russian Academy of Sciences, 111a Kievskaya St., Tomsk, 634012, Russia; Senior Researcher, Nuclear Medicine Department; Cardiology Research Institute, Tomsk National Research Medical Center of the Russian Academy of Sciences, 111a Kievskaya St., Tomsk, 634012, Russia; Cardiovascular Surgeon, Department of Cardiovascular Surgery; Cardiology Research Institute, Tomsk National Research Medical Center of the Russian Academy of Sciences, 111a Kievskaya St., Tomsk, 634012, Russia; Head of the Department of Cardiovascular Surgery; Cardiology Research Institute, Tomsk National Research Medical Center of the Russian Academy of Sciences, 111a Kievskaya St., Tomsk, 634012, Russia; Leading Researcher, Nuclear Medicine Department; Cardiology Research Institute, Tomsk National Research Medical Center of the Russian Academy of Sciences, 111a Kievskaya St., Tomsk, 634012, Russia

**Keywords:** aortic aneurysm, inflammation in the aortic wall, scintigraphy

## Abstract

**The aim of this study** was to develop and test a technique for scintigraphic examination of the thoracic aorta, which allows for visualizing foci of inflammation.

**Materials and Methods:**

The study included 15 patients (median age — 61 [47; 73] years) with aortic dilatation more than 45 mm and thoracic aortic aneurysm who were candidates for surgical treatment. All patients underwent a chest scintigraphy with ^99m^Tc-pyrophosphate 48 h before surgery to identify foci of inflammation in the aortic wall. The new technique included intravenous administration of 370 MBq of a radiopharmaceutical (RP), registration of scintigrams at 3 and 6 h after injection of RP in a tomographic mode combined with X-ray computed tomography. After the image reconstruction, subtraction of the later scintigrams from the early ones was performed, followed by analysis of the final images. The results of scintigraphy were compared with the histological data obtained from intraoperative samples of resected aorta.

**Results:**

According to the results of this novel scintigraphic technique, artifacts from the radioactivity of the vascular blood pool were eliminated and pathological RP uptake was identified in 5 (33.3±1.5%) out of 15 examined patients. The “focus/vessel lumen” ratio averaged at 1.47 [1.30; 1.48]. Histological examination of resected aorta samples confirmed the presence of chronic inflammation in 4 (26.7±1.3%) out of 15 patients. Parameters of diagnostic efficiency were: sensitivity — 100%, specificity — 91%, diagnostic accuracy — 93%.

**Conclusion:**

The method of scintigraphic diagnostics of inflammatory processes in the aorta using ^99m^Tc-pyrophosphate, supplemented by subtraction of the late from the early images, makes it possible to eliminate artifacts from the radioactivity of the aortic blood pool and to reveal the pathological RP accumulation indicating the areas of inflammation in the aortic wall.

## Introduction

According to the European Society of Cardiology (ESC), aneurysm is one of the most common aortic diseases [1]; its occurrence reaches 2–5 cases per 100,000 population per year [2–4]. In half of the patients, pathognomonic symptoms appear only after the thoracic aorta (TA) becomes critically dilated [5]. The main risk posed by TA aneurysms is a high likelihood of developing the acute aortic syndrome — a severe complication that requires immediate surgical intervention [6, 7]. The two-year survival rate for non-operated patients is only 52% [8].

To date, the main indication for urgent surgery is the aortic diameter and its increase over time [1, 9]. It is believed that surgical treatment is justified in patients with an TA enlarged to >50 mm, who do not suffer from hereditary connective tissue diseases [10, 11]. In 30–60% of cases, however, acute aortic syndrome develops in patients with a TA diameter of <50 mm [12–14]; therefore, a search for additional predictors of acute aortic syndrome in patients with TA aneurysm remains relevant. In addition to the morphometric parameters of the aorta, this search must include its morphological and functional characteristics.

Molecular studies and modeling of aortic aneurysms in animals have shown that the early stages of TA dilation are characterized by dysfunction of the endoplasmic reticulum, apoptosis of smooth muscle cells, and inflammation, which aggravates the dilation process [15, 16]. On this basis, we assumed that diagnostic imaging of inflammatory changes in the aortic wall would reveal new predictors of complications associated with a TA aneurysm.

We have proposed methods for scintigraphic diagnosis of inflammation in the heart using the ^99m^Tc-pyrophosphate radiopharmaceutical (RP) with a high diagnostic efficiancy [17–22]. In its original mode, however, these techniques cannot be used to visualize inflammation in the TA wall, since the radioactivity of the blood pooled in cavities of large vessels “overlaps” the local RP radioactivity in adjacent tissues [17–20]. We hypothesized that the adjustment of this technique [23] by subtracting the background radiation, also known as the background correction method, would eliminate artifacts from the radioactivity of the aortic blood pool, improve the quality of visualization of the aortic wall, and identify foci of inflammation, which accumulate ^99m^Tc-pyrophosphate.

**The aim of this study** was to develop and test a technique for scintigraphic examination of the thoracic aorta wall, which allows for visualizing foci of inflammation.

## Materials and Methods

The study included 15 patients (of those — 10 men (66.7±1.5%)) with aortic dilatation (an increase in the maximum diameter of >45 mm). The median age of patients was 61 [47; 73] years. The diagnosis of TA aneurysm was confirmed by computer-aided tomographic (CT) aortography performed in the Cardiology Research Institute, Tomsk National Research Medical Center of the Russian Academy of Sciences. All the patients were considered candidates for surgical treatment.

The clinical characteristics of patients included in the study were as follows:

ischemic heart disease was detected in 7 patients (46.7±1.7%);

arterial hypertension — in 11 (73.3±1.3%);

diabetes mellitus — in 2 (13.3±0.8%);

congenital heart disease — in 3 (20.0±1.1%);

bicuspid aortic valve — in 4 (26.7±1.3%);

non-syndromic aortic diseases — in 6 (40.0±1.6%).

The aortic diameter averaged at 50 [49; 51] mm.

The inclusion criteria were: age — 45–65 years; TA dilatation — 45 mm and more; non-syndromic aorta diseases (idiopathic, familial); a normal aortic valve (two- or tricuspid).

The exclusion criteria were: the left ventricular ejection fraction — less than 50%; myocardial infarction within recent 30 days; stroke — within recent 60 days; heart rhythm disturbances; previous heart or TA surgery; syndromic aortic diseases (Marfan, Ehlers–Danlos, Loeys–Dietz, Turner syndromes); pronounced TA atherosclerosis.

The study was approved by the Ethics Committee of the Cardiology Research Institute, Tomsk National Research Medical Center of the Russian Academy of Sciences and was conducted in accordance with the ethical standards set out in the Declaration of Helsinki (2013).

**Figure 1.**
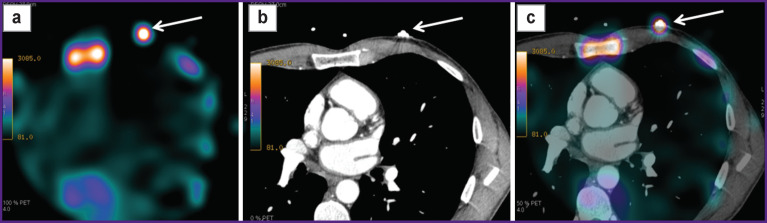
An example of combining scintigraphic and X-ray images using the surface markers: (a) scintigraphic image (axial plane), the arrow indicates the location of the radioactive marker; (b) X-ray tomographic image (axial plane), the arrow indicates the location of the radiocontrast marker; (c) hybrid SPECT/CT image, the arrow indicates the alignment of the both markers

In addition to the standard clinical and instrumental examination, all patients underwent a chest scintigraphy 48 h before surgery. To this end, the RP (^99m^Tc-pyrophosphate) was injected intravenously at a dose of 370 MBq and scintigraphic images were recorded 3 and 6 h later using single-photon emission computer tomography combined with low-dose X-ray CT (SPECT/CT). Sequential images were recorded using a GE Discovery NM/CT 570c hybrid SPECT/CT tomograph (GE Healthcare, USA) equipped with solid-state cadmium-zinc-tellurium detectors and a low-energy multi-pinhole collimator; the measurements were simultaneously made in 19 projections and processed to form a 32×32-pixel matrix. The scanning time was 400–600 s, depending on the patient’s body weight. Immediately before the first examination, a radioisotope marker was attached to the patient’s chest (3^rd^ intercostal space on the left along the mid-clavicular line), on top of which an ECG electrode — a radio-contrast marker — was glued (Figure 1) [23].

After reconstruction, the scintigraphic images were loaded into the Load To New software application (Advantage Workstation 4.6, GE Healthcare). In cell A, the early scintigrams (received after 3 h) were stored, in cell B — the later ones (after 6 h). The frame range was identical for both cells: from the 1^st^ to the 50^th^ frame. The resulting images were obtained by subtracting each frame of cell B from the respective frame of cell A. The resulting images were saved. Alignment of these CT images was performed by precise superposition of the radioisotope and radiocontrast labels in the frontal, sagittal, and transverse planes using the Advantage Workstation 4.6 in the VoluMetrix mode. For a quantitative analysis of the obtained images, the level of RP-generated radiation in various structures of the mediastinum was calculated by automatically counting the number of impulses in the regions of interest (ROI) with the subsequent determination of the “focus/vessel lumen” coefficient.

We defined the “pathological accumulation of ^99m^Tc-pyrophosphate” in the TA wall as the accumulation of RP corresponding to the following conditions: 1) the focus is located in the TA wall (localization was determined by CT); 2) the focus was visually brighter than the background and vessel lumen; 3) quantitatively, the focus-associated RP signal was >1.2-fold higher than the background and radioactivity in the vessel lumen, i.e. the “focus/vessel lumen” ratio was >1.2.

To verify the results of scintigraphy, histological examination of intraoperative material (resected aorta samples) taken from the patients was performed.

Subsequently, the results of SPECT/CT were compared with the morphological data in order to assess the diagnostic efficiancy of the proposed method to diagnose inflammatory processes in the TA.

**Statistical data processing** was performed using the Statistica 10.0 software. Due to the many exclusion criteria and the relatively rare occurrence of diseases meeting the inclusion criteria, the study involved a small number of patients. The normality of data distribution was checked using the Shapiro–Wilk test. Since none of the quantitative data obeyed the normal distribution pattern, the results were presented in the form of the median (Me) and the 1^st^ and 3^rd^ quartiles [Q1; Q3]. Qualitative parameters were presented in the form of absolute numbers (n) indicating the proportions (%) and standard deviation P±σ_P_%.

Indicators of sensitivity and specificity were calculated using the following formulas:


$$\begin{array}{l} \text{sensitivity = TP/(TP + FN)}\cdot \text{100\%;}\\ \text{specificity = TN/(TN + FP)}\cdot \text{100\%;}\\ \text{diagnostic accuracy = (TP + TN)/}\\ \text{/(TN + TP + FN + FP)}\cdot \text{100\%}, \end{array}$$

where TP is a true positive result; TN — true negative result; FP — false positive; FN — false negative result.

Pathological accumulation of ^99m^Tc-pyrophosphate in the TA wall combined with signs of inflammation (as revealed histologically) was taken as TP; for TN — the absence of pathological accumulation of ^99m^Tc-pyrophosphate in the aortic wall in the absence of inflammation; for FP — pathological accumulation of ^99m^Tc-pyrophosphate in the aortic wall in the absence of inflammation; for FN — the absence of pathological accumulation of ^99m^Tc-pyrophosphate in the aortic wall in the presence of histologically confirmed inflammation.

## Results

In histological examination of the resected aorta specimens, foci of chronic inflammation and hemorrhage in the adventitia were detected in 4 (26.7±1.3%) of 15 patients; atheromatosis and parietal thrombosis — in 1 (6.67±1.5%), cystic median necrosis, elasticity disorder, and fibrosis — in all examined patients.

**Figure 2.**
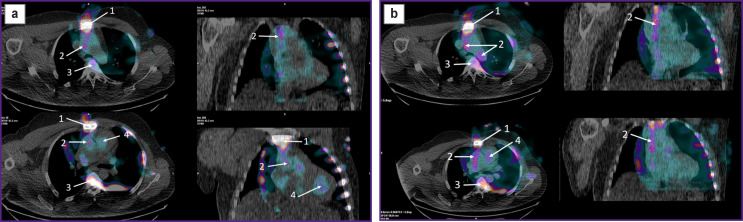
SPECT/CT images of the chest (axial slices — on the left; frontal slices — on the right) of patient P., performed 3 (a) and 6 h (b) after the administration of ^99m^Tc-pyrophosphate The arrows point to the mediastinal structures, which produce significant artifacts and do not allow clearly identifying the pathological accumulation of RP in the ascending and descending aorta walls. *1* — sternum, *2* — radioactive blood pool in the ascending aorta, *3* — spine, *4* — radioactive blood pool in the left ventricle

According to the SPECT/CT data, in both early and late images obtained, respectively, 3 and 6 h after the injection of ^99m^Tc-pyrophosphate, intense radioactivity was detected in the blood pooled in TA cavities; this phenomenon interfered with any attempt to detect radioactivity generated by the aortic wall (Figure 2).

**Figure 3.**
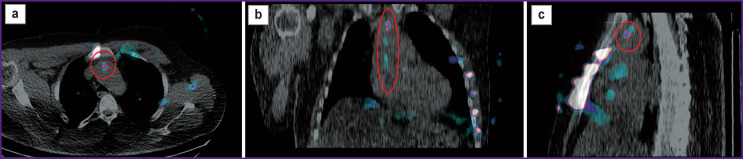
The resulting SPECT/CT images of the chest of patient P. (axial (a), frontal (b), and sagittal (c) slices), obtained after performing subtraction of the baseline images shown in Figure 2 The resulting SPECT/CT images show no significant artifacts from the blood pooled in the large vessels and the heart cavities, as well as from the sternum, which made it possible to visualize pathological focal RP accumulation in the ascending aorta wall (circled in red)

We then applied the subtraction technique, which made it possible to level the interfering factors: the resulting images showed no background or blood-associated radioactivity. Due to this approach, in 5 (33.3±1.5%) patients, foci of pathological RP accumulation in the TA wall were revealed (Figure 3, the Table). The median “focus/vessel lumen” ratio was 1.47 [1.30; 1.48].

Subsequent comparison of the above SPECT/CT results with the histological data confirmed the presence of inflammation in the aortic wall in 4 (80.0±2.0%) of 5 patients with foci of pathological ^99m^Tc-pyrophosphate accumulation (see the Table).

**Table:** Characteristics of resected aorta specimens from patients with pathological aortic accumulation of ^99m^Tc-pyrophosphate (n=5)

Parameter	Patient No.				
	1	2	3	4	5
Localization of ^99m^Тc-pyrophosphate accumulation in the thoracic aorta	Arch	Descending aorta	Arch, descending aorta	Ascending aorta	Ascending aorta
“Focus/vessel” lumen ratio	1.21	1.47	1.30	1.57	1.48
Localization of the thoracic aortic aneurysm (CT scan)	Ascending aorta	Ascending and descending aorta	Ascending aorta	Ascending aorta	Ascending aorta
Histological characteristics of the resected specimen	Cystic median necrosis, fibrosis, foci of chronic inflammation and hemorrhage in the adventitia	Cystic median necrosis, fibrosis, striped foci of chronic inflammation and hemorrhage in the adventitia	Cystic median necrosis, fibrosis, foci of chronic inflammation and hemorrhage in the adventitia, dissection, atheromatosis, parietal thrombosis	Cystic median necrosis, fibrosis, foci of chronic inflammation and hemorrhage in the adventitia	Cystic median necrosis, abnormal elasticity, fibrosis

Among the 15 examined patients, there were four TP results, ten TN results, one FP result, and none FN results. Preliminary indicators of the informative value of the technique were: sensitivity — 100%; specificity — 91%; diagnostic accuracy — 93%.

## Discussion

It is known that aneurysm formation results from an expanding apoptosis–inflammation–remodeling cycle (fragmentation and depletion of fibers), which, together with physical factors, leads to stretching of the aortic wall with the risk of its dissection and rupture. It is accepted by many in the field, that the anatomical characteristics of an aortic aneurysm and the pace of its expansion do not provide enough information to stratify the risks of adverse clinical events. An integrated approach is required to assess the morphological and functional state of the aortic wall. Considering that no histological material from the aortic wall is available before surgery, there is a need to apply non-invasive and highly specific methods for diagnosing the aortic wall inflammation. In this regard, molecular imaging methods namely, positron emission tomography and SPECT, are capable of identifying the inflammatory process at its various stages by using tracers with high and specific affinity to the ROI [24].

Visualization of vascular inflammation in TA is a meticulous and time-consuming job that requires a precise scientific methodology. These technical difficulties are due to the small thickness of the wall in the ascending aorta and the arch, the major presence of large vessels and heart cavities in the area close to the aorta, and the periodic vessel movements due to breathing. These factors cause projection overlays and motor artifacts that interfere with the sought image. Although these difficulties can be eliminated by using multimodal imaging techniques and gating methods, the problem of visualizing the pools of blood located in large vascular cavities has not been fully resolved [17–23].

The main clinical purpose of preoperative non-invasive imaging, in this case, is assessing the degree of inflammation in the aortic wall and, based on that, planning the scope of surgical treatment. This, in turn, can influence the choice of a surgical approach.

In the presented study, the possibility of scintigraphic visualization of inflammation foci in the TA wall using ^99m^Tc-pyrophosphate was demonstrated for the first time. This result was enabled by subtracting the late images from the early ones, which made it possible to neutralize the interference from the blood pooled in the vessels. In the future, the proposed technique can be used with other RPs that are tropic to the inflammatory process, for example, labeled leukocytes, ^201^Tl, etc. The validity of the results was confirmed by the histological examination. In this study, only one false positive result was obtained: the detected accumulation of RP in the aorta wall was not confirmed by histological data. This could be due to the discrepancy between the area of actual inflammation and the area of histological examination of the resected sample. The preliminary indicators of diagnostic accuracy obtained in the presented study were high, but, given the small number of patients, the data need further confirmation. The absence of a control group is also a limitation of the presented study.

## Conclusion

Scintigraphic diagnosis of inflammation in the patients’ aorta using ^99m^Tc-pyrophosphate, supplemented by subtracting the late from the early images, makes it possible to eliminate artifacts from the radioactivity generated by aortic blood pools and to detect the foci of pathological radiotracer accumulation revealing the areas of inflammation in the aortic wall.
